# Retraction: Exercise, circadian rhythms, and muscle regeneration: a path to healthy aging

**DOI:** 10.3389/fnins.2026.1967875

**Published:** 2026-08-19

**Authors:** Frontiers Editorial Office

The journal retracts the 09 October 2025 article cited above.

Following publication, concerns were raised regarding the integrity of the images in the published figures.

The authors failed to provide a satisfactory explanation during the investigation, which was conducted in accordance with Frontiers' policies. As a result, the data and conclusions of the article have been deemed unreliable and the article has been retracted.

This retraction was approved by the Chief Editors of Frontiers in Neuroscience and the Chief Executive Editor of Frontiers. The authors received a communication regarding the retraction and had a chance to respond. This communication is recorded by the publisher.

Frontiers would like to thank the concerned reader who contacted us regarding the published article.

